# Exploring the geography of serious mental illness and type 2 diabetes comorbidity in Illawarra—Shoalhaven, Australia (2010 -2017)

**DOI:** 10.1371/journal.pone.0225992

**Published:** 2019-12-05

**Authors:** Ramya Walsan, Darren J. Mayne, Nagesh Pai, Xiaoqi Feng, Andrew Bonney

**Affiliations:** 1 School of Medicine, Faculty of Science, Medicine and Health, University of Wollongong, Wollongong, Australia; 2 Illawarra Health and Medical Research Institute, University of Wollongong, Wollongong, Australia; 3 Public Health Unit, Illawarra Shoalhaven Local Health District, Warrawong, Australia; 4 The University of Sydney, School of Public Health, Sydney, Australia; 5 Mental Health Services, Illawarra Shoalhaven Local Health District, Wollongong Hospital, Wollongong, Australia; 6 Population Wellbeing and Environment Research Lab (PowerLab), School of Health and Society, Faculty of Social Sciences, University of Wollongong, Wollongong, Australia; 7 School of Public Health and Community Medicine, University of New South Wales, Sydney, Australia; Johns Hopkins Bloomberg School of Public Health, UNITED STATES

## Abstract

**Objectives:**

The primary aim of this study was to describe the geography of serious mental illness (SMI)–type 2 diabetes comorbidity (T2D) in the Illawarra-Shoalhaven region of NSW, Australia. The Secondary objective was to determine the geographic concordance if any, between the comorbidity and the single diagnosis of SMI and diabetes.

**Methods:**

Spatial analytical techniques were applied to clinical data to explore the above objectives. The geographic variation in comorbidity was determined by Moran’s I at the global level and the local clusters of significance were determined by Local Moran’s I and spatial scan statistic. Choropleth hotspot maps and spatial scan statistics were generated to assess the geographic convergence of SMI, diabetes and their comorbidity. Additionally, we used bivariate LISA (Local Indicators of Spatial Association) and multivariate spatial scan to identify coincident areas with higher rates of both SMI and T2D.

**Results:**

The study identified significant geographic variation in the distribution of SMI–T2D comorbidity in Illawarra Shoalhaven. Consistently higher burden of comorbidity was observed in some urban suburbs surrounding the major metropolitan city. Comparison of comorbidity hotspots with the hotspots of single diagnosis SMI and T2D further revealed a geographic concordance of high-risk areas again in the urban areas outside the major metropolitan city.

**Conclusion:**

The identified comorbidity hotspots in our study may serve as a basis for future prioritisation and targeted interventions. Further investigation is required to determine whether contextual environmental factors, such as neighbourhood socioeconomic disadvantage, may be explanatory.

**Implications for public health:**

Ours is the first study to explore the geographic variations in the distribution of SMI and T2D comorbidity. Findings highlight the importance of considering the role of neighbourhood environments in influencing the T2D risk in people with SMI.

## Introduction

Research has established that type 2 diabetes (T2D) often co-occurs with serious mental illness (SMI) such as schizophrenia, bipolar disorder and major depression[1]. People with SMI have 2–4 times higher risk of developing T2D compared with the general population, which translates into an average reduction of 15–20 years in their life expectancies[2,3]. In contrast, several lines of evidence also suggest that a diagnosis of T2D can increase the risk of mental disorders such as depression[4]. For people with SMI, a comorbid diabetic diagnosis not only confers a higher cardiovascular risk and increased risk of premature mortality, but is also associated with greater cognitive decline, worse prognosis, increased hospitalisations, greater number of emergency department visits, non-adherence to treatments, higher healthcare utilisation costs and decreased quality of life for people experiencing mentally ill-health[3,5–9].

Significant geographic inequalities have been reported in the distribution of both severe mental illness and T2D [10–18]. However, to the best of our knowledge, geographic variations in their comorbidity have not been previously explored. A recent systematic literature review reported a paucity of research literature investigating the association between neighbourhoods and SMI -T2D comorbidity[19]. Moreover, in recent years, there has been increased interest in addressing comorbid conditions concurrently rather than as separate diseases and an integrated management approach is now considered superior over a single focus approach[20]. Exploring neighbourhood variations in the co-occurrence and clustering of SMI-T2D may help us to better understand the overlapping prevalence of these two chronic diseases and to propose novel hypotheses regarding the neighbourhood level factors that might influence the co-occurrence. Describing the geography may also assist public health authorities to cost-effectively target local resources and preventive interventions to reduce the regional disparities and public health burden imposed by the comorbidity.

Accordingly, the purpose of this study was to examine the neighbourhood level geographic variations in SMI-T2D comorbidity, in an Australian community using cross-sectional, routinely collected clinical data. We also aimed to determine the geographic concordance if any, between the comorbidity and the single diagnosis of SMI and T2D.

## Research design and methods

### Study area and population

This cross sectional study was carried out in the Illawarra and Shoalhaven regions of New South Wales, Australia, which had an estimated resident population of 368,604 people at the time of the 2011 Australian Census of Population and Housing[21]. Serious mental illness and diabetes comorbidity data for the period of 2010 to 2017 were obtained from the Illawarra Health Information Platform (IHIP), which is a research partnership established between Illawarra Shoalhaven Local Health District (ISLHD) and University of Wollongong for the purpose of providing ISLHD health service data to researchers. Community-derived diabetes data (without reference to comorbidities), were retrieved from the Southern IML Research (SIMLR) study database for the period of 2010 to 2014. SIMLR is a longitudinal, community-derived near-census database consisting of routinely collected pathology results for residents 18 years and over in Illawarra Shoalhaven[14]. All the data used in this study were deidentified prior to extraction, consistent with the requirements of the Privacy Act 1988 (Cth) and Health Records and Information Privacy Act 2002 (NSW). Residential suburbs were the smallest geographical units at which health service data were available and were used as the spatial units of analysis. Information on the population of the region by age groups and gender was obtained from the 2011 Australian Census of population and housing[21]. To display and analyse the geographic distribution of SMI, T2D and their comorbidity, a base map of the Australian suburbs 2011 digital boundaries from Australian Bureau of Statistics was used. This study was approved by The University of Wollongong and Illawarra Shoalhaven Local Health District Human Research Ethics Committee (protocol number 2017/428).

### Study sample

Serious mental illness in our study was defined as a primary or secondary diagnosis of SMI from the inpatient records of ISLHD. Data extraction was carried out by means of International Classification of Diseases (ICD) 10 codes (Table 1). Comorbidity was defined as having a T2D stay diagnosis code (ICD code E11) in people with serious mental illness recorded in the ISLHD data. Comorbidity details were extracted as either present or absent along with each of the SMI records. The community-derived diabetes sample, consisted of individuals with at least one HbA1c test between 2010 and 2014 and an HbA1c result ≥ 6.5% or plasma glucose levels ≥7.0mmol/L within 12 months of an HbA1c test, consistent with thresholds used in the Australian National Health Measures Survey [22]. Data analysis was restricted to individuals 18 years and over.

**Table 1:** SMI diagnosis groups and ICD 10 codes included in the study.

Diagnosis	ICD 10 codes
Schizophrenia	F20
Other non-affective psychosis	F22 –F29
Bipolar disorder	F30, F31
Major depression	F32, F33
Other affective disorders	F34, F39

### Statistical analysis

We calculated the relative risk of SMI-T2D comorbidity for each of the 167 suburbs in the Illawarra Shoalhaven region by computing the ratio of observed to the expected counts. The expected number of cases was calculated by indirect standardisation and was obtained by multiplying age-sex stratified population in each suburb by the age-sex stratified prevalence across the entire study area. Expected counts for males and females aged 18–34, 35–49, 50–64 and 65+years were calculated separately and were then aggregated within suburbs to create an aggregated denominator for the relative risk. Data over the entire study period (2010–2017) were combined to ensure sufficient counts. The population profile of the study area had remained relatively similar during these time period[21]. Suburbs with expected counts of zero (n = 5) were merged with the neighbouring suburbs for further analysis. Large variance in relative risks was observed due to sparse comorbidity counts and the heterogeneous population density in the area. To address this issue, relative risk data were smoothed using the Empirical Bayes smoothing technique recommended by Anselin and Koschinsky to shrink and stabilise the rates towards the global mean of the whole study region[23].

Global Moran’s I was used to investigate spatial autocorrelation or clustering in the raw estimates[24]. Moran’s I statistic ranges between -1 and 1, with a value of zero indicating complete spatial randomness; a positive value indicating positive spatial autocorrelation; and a negative value indicating negative spatial autocorrelation[24]. Local Indicator of Spatial Association (LISA) and spatial scan statistics were used to identify the location of comorbidity clusters. These two spatial analytical techniques were adopted simultaneously to complement the findings and to provide more intuitive results [25]. LISA, often known as Local Moran’s I, was used to detect the significant clusters of higher and lower relative risks of comorbidity[24]. High-high clusters are areas of significantly high rates surrounded by other areas of significantly higher rates, and low-low clusters represents areas with lower risks surrounded by other areas of lower values[26,27].

Spatial scan statistics works by imposing circular scanning windows of varying radii, which gradually moves over the study area evaluating the likelihood ratios of all potential clusters using a user defined maximum percentage of population at risk[28], which in this analysis was set at a default maximum spatial cluster size of ≤ 50%[29]. We employed a purely spatial retrospective scan using the discrete Poisson model, where by the number of events is assumed to be Poisson distributed [28]. The input data for this model consisted of the observed and the expected comorbidity counts. The ‘no geographic overlap’ criterion was used to report the clusters and the p values calculated were two tailed.

In order to compare the geographic concordance of SMI-T2D comorbidity with the single diagnosis of SMI and diabetes in the general population (Gen-DM), relative risk maps, LISA maps and spatial scan statistics were generated for SMI and Gen-DM following the same procedures as the comorbidity map. Additionally, we used bivariate LISA[26,27] and multivariate spatial scan[28] statistics to test the association between SMI and Gen-DM and to map their associations at suburb level. The LISA bivariate statistic indicates how observations of a variable (SMI) in a certain suburb are associated with the observations of a different variable (Gen-DM) in the adjacent suburb. In our case, high-high clusters will indicate coincident areas of high rates of SMI and Gen-DM and low-low clusters will be the areas of coincident low rates of SMI and Gen-DM. Multivariate spatial scan identifies spatial clusters with higher and lower rates for both SMI and Gen-DM by simultaneously searching for and evaluating clusters within the two datasets. The likelihood ratio for each data set is summed up to determine the likelihood ratio for that particular window[28].

The statistical significance of Global Moran’s I, Local Moran’s I, Spatial scan and bivariate LISA were evaluated under the complete spatial randomness assumptions using 9999 Monte Carlo simulations and a significance level of 0.05[30]. Benjamini Hochberg correction was applied to control for false discovery rates in LISA and Bivariate LISA statistics[31].

### Software

We used Geo Da [27] for Empirical Bayes Smoothing and spatial analysis, SaTScan for univariate and multivariate spatial scan statistics[28], R for descriptive analysis [32] and Arc GIS 10.5 for mapping[33].

## Results

### Sample description

A total of 4165 unduplicated records were extracted with an SMI diagnosis between 1 January 2010 and 31 December 2017. Individuals residing outside the Illawarra Shoalhaven area (n = 50) and records with no suburb information (n = 283) were excluded from our analysis (n = 341, 8.2%) resulting in a final SMI sample of 3824 people. Of these, 463 (12.1%) had a T2D comorbidity. The community-derived diabetes sample for the region consisted of 13142 unique individuals. The distribution of SMI, diabetes and their comorbidity in the Illawarra and Shoalhaven is described in Table 2. The median age of the comorbidity subgroup was 58 years (range = 18–92 years). The gender distribution was approximately equal with females accounting for 52.9% of the sample. Higher comorbidity prevalence was observed in older adults above 50 years of age.

**Table 2:** Distribution of serious mental illness, type 2 diabetes and their comorbidity in Illawarra Shoalhaven (2010–2017).

Demographic characteristics	Serious mental illness	Diabetes	Serious mental illness -type 2 diabetes comorbidity
Total	3824	13142	463
**Sex**			
**Male** **n (%)**	1977 (51.7)	7248 (55.2)	218 (47.1)
**Female** **n (%)**	1847 (48.3)	5894 (44.8)	245 (52.9)
**Age (Years)**			
**18–34** **n (%)**	1132 (29.6)	189 (1.4)	27 (5.8)
**35–49** **n (%)**	1220 (31.8)	733 (5.6)	108 (23.3)
**50–64** **n (%)**	820 (21.4)	3294 (25.1)	150 (32.4)
**65+** **n (%)**	652 (17.1)	8926 (67.9)	178 (38.4)

### Spatial distribution of SMI -T2D comorbidity

**Fig 1:**
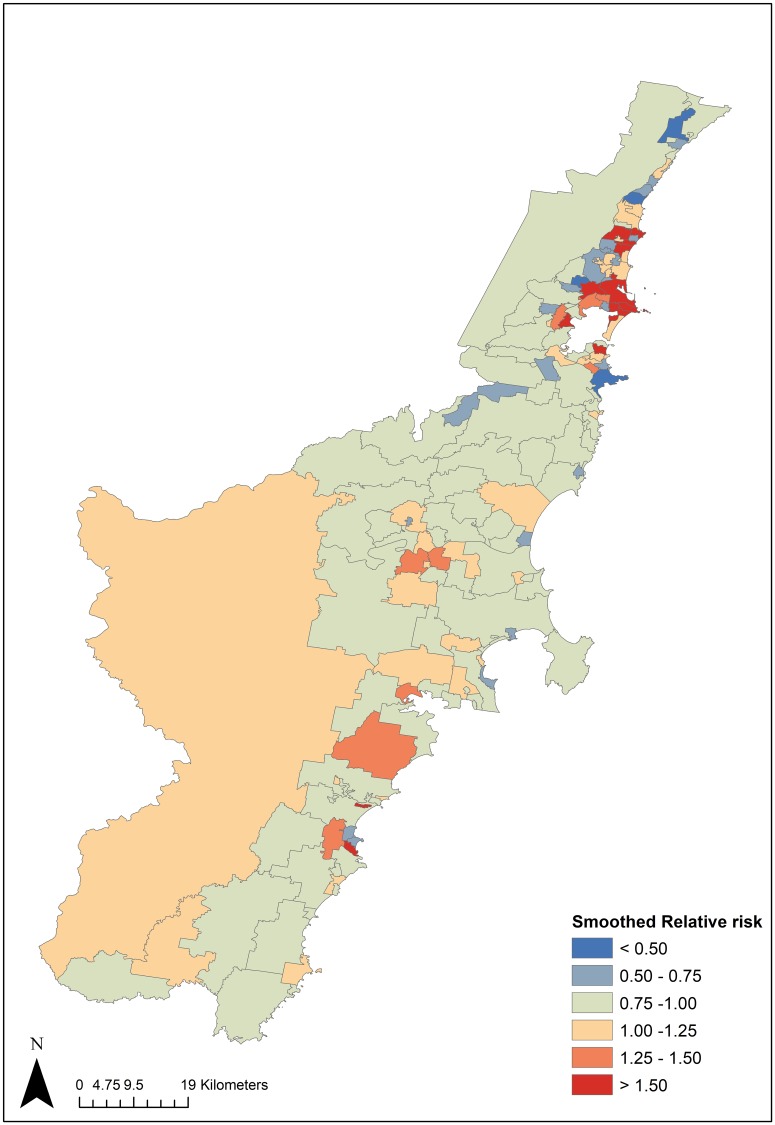
Smoothed relative risk of SMI-T2D comorbidity in the Illawarra Shoalhaven (2010–2017).

**Fig 2:**
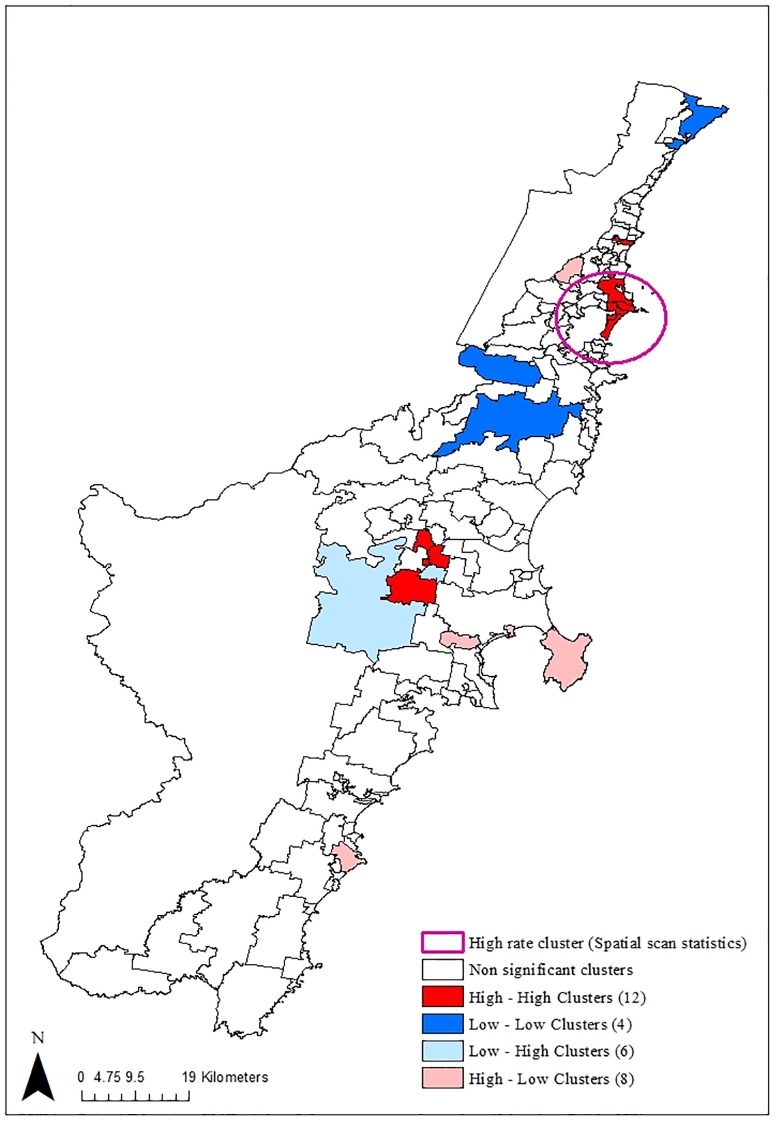
Local Moran’s I and spatial scan statistics calculated for SMI-T2D comorbidity in Illawarra—Shoalhaven (2010–2017).

The geographic distribution of smoothed relative risks for SMI-T2D comorbidity in the Illawarra and Shoalhaven is depicted in Fig 1. Moran’s I revealed a positive global spatial autocorrelation for SMI-T2D relative risk (Moran’s I = 0.1155, p = 0.0361) indicating that suburbs with similar SMI- T2D risk are clustered geographically. Fig 2 demonstrates the results of the application of LISA and spatial scan statistics to the SMI-T2D comorbidity risk by suburbs.

LISA analysis identified twelve (12) significant high-high clusters (hotspots) and four (4) low-low clusters (cold spots), that became non-significant after correcting for multiple comparisons using the Benjamini–Hochberg FDR procedure. However, there was a strong correspondence between uncorrected LISA and spatial scan analysis in identifying a hotspot south of major metropolitan area as shown in Fig 2. The spatial scan statistics using a maximum cluster size of ≤50% of total population identified one significant high rate cluster of SMI-T2D comorbidity in the suburbs south of major metropolitan city centre (Fig 2). The high rate cluster identified comprised of 23 urban suburbs and had a relative risk of 1.80 (p <0.001). The number of observed comorbidity cases in this cluster was 110, compared to 68 expected cases. The identified high rate cluster contained 14.2% of the total population in the Illawarra Shoalhaven. No significant low rate clusters were detected by spatial scan. Six urban suburbs south of major metropolitan city were identified as high-risk areas for SMI-T2D comorbidity as they consistently appeared in both LISA and spatial scan statistics as a high rate cluster.

### Geographic concordance of SMI, T2D and their comorbidity

**Fig 3:**
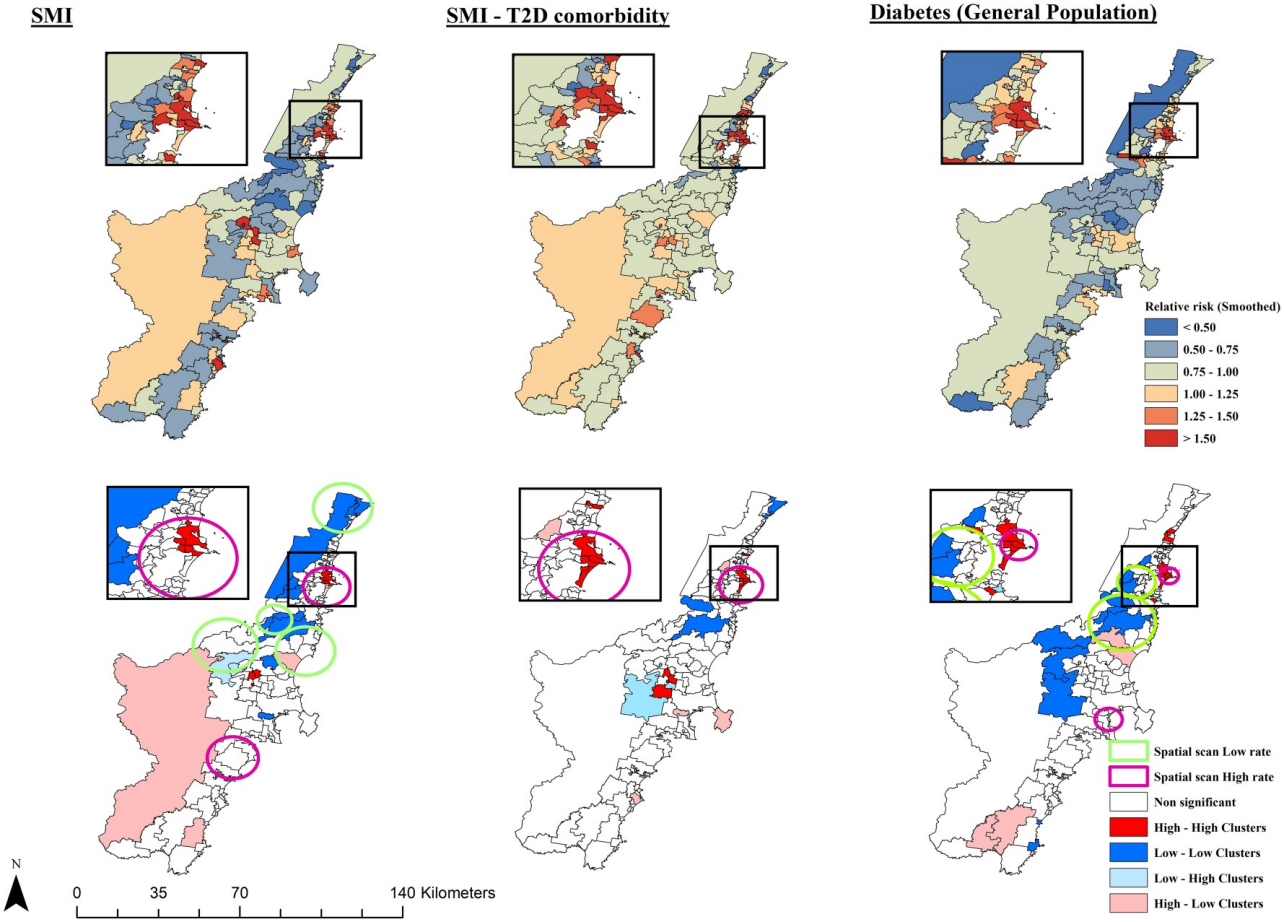
Geographic distribution and significant hotspots for SMI, diabetes and SMI -T2D comorbidity in Illawarra—Shoalhaven (2010–2017).

In order to compare the geographic concordance of SMI-T2D comorbidity with the SMI and diabetes risk in Illawarra Shoalhaven, smoothed relative risk maps, LISA maps and spatial scan statistics were generated for SMI, Gen-DM and SMI-T2D comorbidity (Fig 3). For SMI, we identified 6 high-high clusters, 10 low-low clusters, 4 low-high clusters and 8 high-low clusters. For Gen-DM the high-high, low-low, low-high and high-low clusters identified were 6, 12, 2 and 5 respectively. Both LISA and spatial scan statistics (Table 3) consistently identified a convergence of hotspots (high-high clusters) for SMI, T2D and their comorbidity in four urban suburbs south of the major metropolitan centre, which was previously identified as a comorbidity hotspot.

**Table 3:** Significant spatial scan clusters of SMI, diabetes (general population) and SMI—T2D comorbidity (Illawarra -Shoalhaven 2010–2017).

Diagnosis	Cluster Type	No of suburbs	Observed count	Expected Count	Relative risk	Log likelihood	P value
SMI	High	24	1350	1056.13	1.43	53.54	<0.001
	High	12	222	152.37	1.49	14.58	<0.001
	Low	16	248	404.99	0.59	38.89	<0.001
	Low	3	1	26.21	0.038	22.02	<0.001
	Low	5	31	60.43	0.094	14.53	<0.001
SMI-T2D comorbidity	High	23	163	102.97	1.89	20.02	<0.001
Gen-DM	High	4	917	577.09	1.63	89.40	<0.001
	High	5	1157	967.84	1.21	18.86	<0.001
	Low	14	1076	1555.69	0.66	92.80	<0.001
	Low	12	570	732.79	0.77	20.65	<0.001

**Fig 4:**
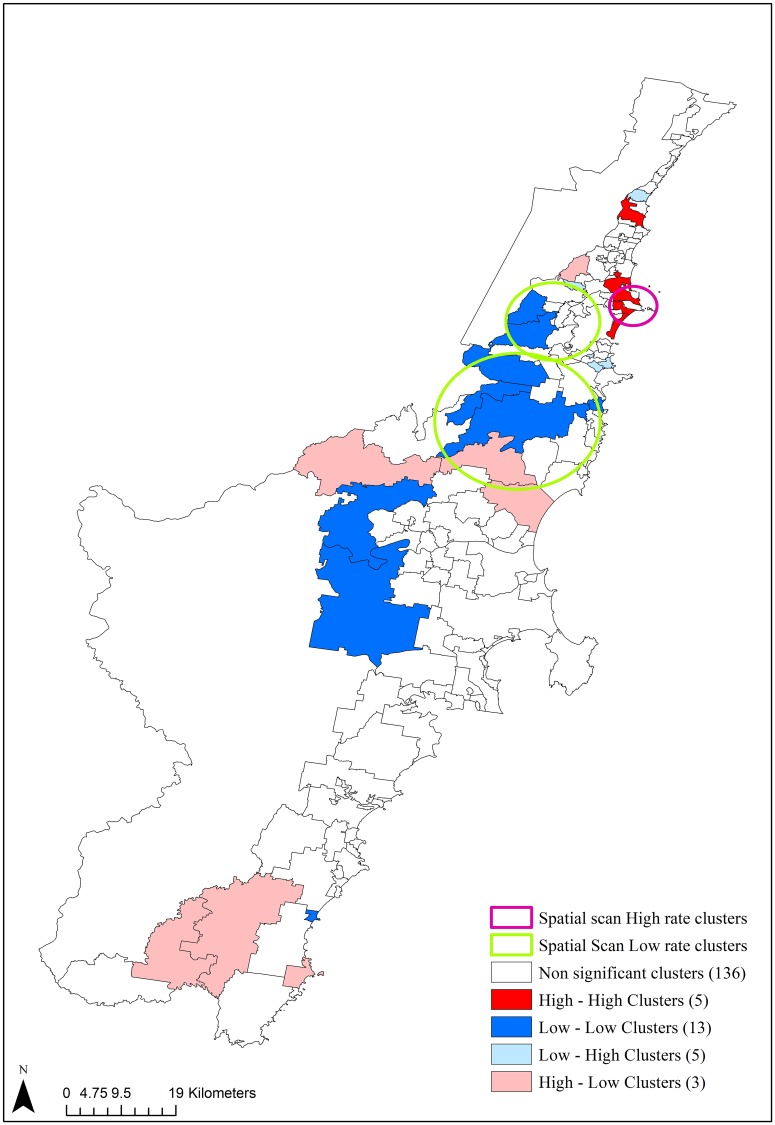
Bivariate LISA and multivariate spatial scan clusters showing the local association between SMI and diabetes in Illawarra and Shoalhaven (2010–2017).

Fig 4 shows the result of bivariate LISA analysis and multivariate spatial scan for SMI and diabetes in Illawarra and Shoalhaven. Five high-high clusters indicating suburbs of higher SMI risk surrounded by neighbourhoods of higher diabetes risk were observed in the southern urban areas. The analysis also revealed 7 low-low clusters in the central part of study region. Similar to LISA clusters, application of multiple comparison correction to these results didn’t yield any significant results. Multivariate spatial scan analysis with a maximum spatial cluster size of up to 50% identified one high rate cluster for both SMI and Gen-DM comprising of 4 suburbs with a relative risk of 1.63 (log likelihood ratio 178.8, p <0.001).

## Discussion

The present study identified geographic variations in the distribution of SMI–T2D comorbidity in the Illawarra Shoalhaven. The spatial dependence of comorbidity was confirmed by the global test for spatial autocorrelation (Moran’s I). In other words, suburbs with higher comorbidity risk tend to locate closer than we would expect at random. Conversely, suburbs with lower comorbidity risk also tend to cluster together geographically. Using local indicators of spatial association (LISA and spatial scan statistics), we were able to identify a consistently higher burden of comorbidity in Six urban suburbs south of the metropolitan city. These suburbs are relatively homogeneous in terms of their population density and socioeconomic environments. Comparison of comorbidity hotspots with the hotspots of single diagnosis SMI and diabetes further revealed a geographic concordance of high-risk areas in four urban regions of the main metropolitan. These findings suggest that the population in some urban suburbs are challenged by SMI, T2D and their comorbidity and appropriate prevention and management initiatives should be targeted accordingly. This study has also demonstrated the potential usefulness of combining spatial analytical methods and clinical data information to inform health service commissioning and geographically target needs-based preventive interventions.

We observed that both LISA and bivariate LISA clusters became non-significant after correcting for multiple comparisons using Benjamini Hochberg procedure. Even though Benjamini–Hochberg correction is a less conservative method compared to other false discovery correction procedures, there can still be substantial loss of power (constraining the type I error rate at the expense of an increasing type II error rate) when dealing with bigger datasets [31]. This loss of power could have contributed to our null results. Despite this potential limitation, the correspondence between uncorrected LISA and spatial scan analyses in identifying hotspots south of the major metropolitan area indicate that our results remain interesting. This is the first study to explore the geographic variations in the distribution of SMI and T2D comorbidity. Lack of evidence in this important area of public health was highlighted in a recent systematic literature review[19]. Previous research has, however, established significant geographic inequalities and urban clustering in the distribution of both SMI and type 2 diabetes[13–18]. In this study, we were able to demonstrate that this relationship holds true for their comorbidity as well. From a health service research and policy perspective, describing the geography of coexisting diseases together might prove more useful in aiding decisions on the allocation of resources and integrated interventions. Findings from this study will also create opportunities for further exploratory hypothesis testing, using spatial clustering as a framework. One commonly hypothesised and plausible contributory exposure is neighbourhood socioeconomic disadvantage. Disadvantaged neighbourhoods often expose mentally ill persons to greater psychosocial stress, or act as a proxy for adverse health behaviours such as unhealthy eating, lack of physical activity and obesity, which have been shown to be associated with increased T2D risk[17,34,35]. Thus, identification and exploration of these neighbourhood features that might influence SMI -T2D comorbidity will be an important next step for enhancing our understanding of the geography of comorbidity and will be addressed in future research.

The overall aim of our study was to generate information that could be useful to guide health service policies and preventive interventions aimed at reducing the burden of T2D comorbidity in people with serious mental illness. We have identified hotspots of SMI, T2D and their comorbidity in some urban regions of the Illawarra–Shoalhaven. Targeted health care strategies focussed on these regions may possibly reduce the health inequality and public health burden imposed by SMI–T2D comorbidity.

The results from this study should be interpreted with respect to their limitations. Firstly, the serious mental illness and comorbidity data were sourced only from inpatient mental health records of ISLHD and did not consider outpatient and private practice records. Even though this is supported by the data from the Australian National Surveys of Psychosis (indicating that 45.6–62.9% of people with SMI reported ≥1 hospital admission for any reason in the previous 12 months)[36], the results from our study cannot be applied to the general population. The second limitation is the cross-sectional study design that does not permit cause and effect conclusions. We also note that there’s a potential for temporal misalignment as 2011 census data was used as the reference population. However, a sensitivity analysis using 2016 census data did not alter the results significantly.

### Conclusions

In this study we combined spatial analytical methods and clinical data to analyse the spatial distribution of SMI -T2D comorbidity in Illawarra Shoalhaven. Our results revealed evidence of spatial variations in the distribution of SMI -T2D comorbidity. The high-risk clusters were mainly located in the urban areas. The findings from this study emphasise the geographic focus needed in these regions to reduce the T2D burden in SMI. This study has also demonstrated the potential of spatial analytical methods in assessing and identifying spatial disparities in the comorbid disease risks so that preventive interventions and resources are appropriately targeted. Further investigation using multilevel analytical techniques is required to determine whether particular environmental factors such as neighbourhood socioeconomic disadvantage may be explanatory for these geographic variations in SMI -T2D comorbidity. Understanding the neighbourhood correlates will help us in developing evidence based holistic interventions, health care policies and potentially the design of healthier places to live.
